# Probiotic Viability Reconsidered: Integrating VBNC Resuscitation and Culture-Independent Methods for Accurate Probiotic Enumeration

**DOI:** 10.3390/microorganisms13112479

**Published:** 2025-10-30

**Authors:** Sara Arroyo-Moreno, Gonzalo Saiz-Gonzalo, Seamus McSweeney, Sinead B. Bleiel

**Affiliations:** AnaBio Technologies, T45 RW24 Carrigtwohill, Co. Cork, Ireland; gonzalo.saiz@anabio.ie (G.S.-G.); seamus.mcsweeney@anabio.ie (S.M.)

**Keywords:** probiotics, VBNC, flow cytometry, functional foods, encapsulation

## Abstract

Probiotic enumeration in foods and beverages remains anchored in culture dependent colony-forming unit (CFU) counts, the regulatory gold standard for label compliance. However, culturability does not fully equate to viability as environmental stresses can convert probiotic cells into a viable but non-culturable (VBNC) state, where they remain metabolically active but undetectable by CFU counts. Microencapsulation can provide a degree of protection to probiotics against stress; nevertheless, this blind spot in quantification forces manufacturers to overdose formulations or risk non-compliance with health benefits claims. Thus, the efficacy of probiotics may be underestimated when evaluation relies solely on CFU, creating a false dichotomy between VBNC and non-viable cells. Culture-independent methods, including flow cytometry quantification of active fluorescent units (AFUs), viability PCR/dPCR, and rRNA-targeted Flow-FISH, can aid closing this gap by detecting metabolically active cells non-detectable by culturing, providing complementary quantification data to CFU counts alone. Understanding the relationship between quantification by culture and culture-independent methods provides a more accurate measure of probiotic dose delivery in functional foods and beverages. This review covers the current understanding of VBNC state, including induction, detection, and resuscitation in probiotics, with emphasis on experimental controls that differentiate true VBNC resuscitation from population growth. Case studies in *Lactobacillus* and *Bifidobacterium* illustrate triggers, molecular mechanisms, and methodological advances. Finally, guidance is provided for the development of an integrated quantification approach that reconciles culture-dependent and culture-independent data, ultimately aiming to improve CFU count accuracy through the controlled resuscitation of VBNC cells.

## 1. Introduction

Probiotics are defined as live microorganisms that confer a health benefit on the host (FAO/WHO/ISAPP consensus) when administered in adequate amounts. Most probiotic-containing products deliver a daily level of 10^9^ live probiotics. Health benefits, supported by evidence, include a reduction in antibiotic-associated diarrhea, modulation of the gut microbiota, and support of the immune function [1]. Despite their broad use in foods and supplements, probiotic viability is compromised across the product life cycle by exposure to multiple stresses, from manufacturing (e.g., extreme temperature, high pressure) and storage (e.g., temperature fluctuations) to GI tract transit (e.g., low pH, bile salts) [2]. One adaptive strategy to survive such stresses is entry into the viable but non-culturable (VBNC) state, in which cells remain alive but fail to grow on standard media [3,4,5]. Encapsulation technologies using protein or polysaccharide-based matrices are widely employed to enhance probiotic survival in foods and beverages, by preserving membrane integrity and buffering stresses [6,7]; however, since VBNC fractions are not detected by CFU counts, the protection provided by encapsulation is often underestimated.

Bacteria in the VBNC state typically maintain membrane integrity, basal metabolism, and rRNA/ATP signatures, while showing changes in cell morphology such as reduced size, slowed metabolism, altered gene expression, and increased envelope rigidity [4,8]. Additional features often include active efflux and retained enzymatic activities and increased envelope rigidity through changes in cell wall/membrane composition, which can be proven with viability stains and functional assays. Therefore, bacterial cells in the VBNC state are not strictly dormant, being able to sense environmental cues and, under favorable conditions, resuscitate to regain their culturability [9].

Resuscitation denotes the recovery of colony-forming ability by previously non-culturable cells without requiring cell division, whereas growth entails replication that increases population size [6,10]. Distinguishing these phenomena is critical because conventional plate counts miss VBNC cells and can underestimate true viable probiotic numbers in foods and supplements [11].

Robust evidence for resuscitation, rather than the regrowth of a few surviving cells, requires demonstrating a clear distinction between total viable counts and colony-forming units (CFUs). Specifically, total counts measured by culture-independent methods should remain stable while CFUs increase, or recovery should occur only under specific rescue conditions (e.g., antioxidants) but not on routine media [12,13]. Known triggers for reversing the VBNC state include nutrient upshifts, temperature and pH adjustments, oxidative stress scavenging (e.g., by catalase, sodium pyruvate), and signaling molecules such as resuscitation-promoting factors [4].

Accurate enumeration and viability assessment of probiotics is therefore moving beyond CFU alone toward culture-independent methods that quantify both culturable and VBNC populations, including ISO-standardized flow cytometry, viability PCR/dPCR, and hybrid Flow-FISH for strain or species level discrimination. These advances align with evolving pharmacopeial and industrial practices and are relevant for regulatory contexts where labels must guarantee viable dose through end of shelf life, therefore intensifying the need for accurate probiotic enumeration for appropriate health claims [8,14].

Encapsulation has been widely adopted to enhance probiotic survival during processing and storage, but verifying its protective effect requires precise viability measurements using culture independent methods to measure truly live fractions [15,16]. However, these methods cannot fully replace the traditional plate count method, as most international regulations still require enumeration by CFU for label compliance. In this light, it is important to understand the level of agreement between culture-dependent and culture-independent methods to quantify live probiotics [17,18].

This review structures operational and quantitative criteria that distinguish viable but non-culturable (VBNC) cell resuscitation from normal growth in lactic acid bacteria (LAB) and probiotics. It also provides practical guidance on media formulations and culture conditions that promote resuscitation before CFU enumeration. Furthermore, the review introduces a dual-metric framework that combines standardized flow cytometry with resuscitation-enhanced CFU counting to bridge culture-independent and culture-dependent enumeration approaches. Finally, it highlights current validation gaps and discusses regulatory considerations to support the standardization of VBNC detection methods.

## 2. VBNC Resuscitation in LAB Strains: Case Studies in the Food and Beverage Industry

VBNC states have been reported in a range of lactic acid bacteria (LAB) exposed to processing, storage, and environmental stresses encountered in the food and beverage industry. The nature of stress, physiological response, and the conditions required to restore culturability vary according to species, strain, and matrix composition [19,20]. Microencapsulation of probiotics can attenuate some of the processing stresses such as thermal shock or oxidative stress, ensuring that cells retain membrane integrity; however, cells still may lose culturability and enter the VBNC state [21,22]. In this section, case studies of LAB commonly found in the dairy and brewing industries are presented, with emphasis on VBNC induction, evidence confirming viability despite loss of culturability, and resuscitation mechanisms. These are summarized in Table 1.

### 2.1. Levilactobacillus brevis, Lactobacillus plantarum, Fructilactobacillus (Lactobacillus) lindneri, and Lactobacillus acetotolerans (Oxidative Stress in Beer and Catalase Resuscitation)

*Lactobacillus brevis* is a species of lactic acid bacteria frequently isolated from beer, where it can play a role in both desirable fermentation processes and spoilage. *L. brevis* enters a VBNC state after a prolonged cold storage and repeated exposure to hop acids, yielding false negatives on routine agar despite retained viability [23]. In the case of *L. brevis* strain HRB 01, a VBNC state was induced by cold storage (0 °C), resulting in it failing to form colonies on MRS agar. Colony recovery was observed when agar was supplemented with catalase (1000 IU/mL) [23]. The resuscitation effect of catalase suggests that peroxide/ROS stresses during cold temperature may have induced *L. brevis* cells to enter the VBNC state [23].

*Lactobacillus plantarum* is another beer-associated bacterium, with roles ranging from spoilage to controlled sour beer production. *L. plantarum* entered a VBNC state following 6 months storage at 4 °C or thirty serial passages in beer, remaining metabolically active yet non-culturable on standard agar [24]. Supplementing recovery media with catalase restored its colony formation ability, establishing oxidative stress relief as a practical resuscitation mechanism [24]. Similarly, cold storage of beer induced a VBNC state in *F. lindneri* (formerly *Lactobacillus lindneri*) and *L. acetolerans*, with resuscitation occurring only on catalase-supplemented agar and spoilage capability restored thereafter [25,26].

### 2.2. Lacticaseibacillus paracasei (Dairy Culture and Nutrient Uptake)

In the yoghurt starter *L. paracasei* Zhang, low temperature and acidity induced a VBNC state. RNA sequencing analysis revealed transcriptional shifts favoring substrate-use efficiency and stress tolerance over replication-associated genes, consistent with a viable but non-growing phenotype [27]. When flow cytometry-sorted VBNC cells were transferred to nutrient-rich MRS, culturability was restored only after an extended lag. In contrast, skim milk supplemented with yeast extract supported limited recovery, representing an operational marker of resuscitation prior to growth [27]. Complementary single-cell Raman spectroscopy revealed molecular composition shifts in VBNC *L. paracasei* Zhang that normalized upon recovery, supporting a “repair then divide” sequence [27].

### 2.3. Bifidobacterium spp. (Acid Stress in Fermented Dairy)

During the storage of yoghurt, it was reported that culturable counts of *Bifidobacterium* spp. declined while cells conserved membrane integrity and rRNA reservoirs, indicating a VBNC state [28,29]. An rRNA stability quantification study showed that dead acid-killed controls rapidly lost their cellular rRNA, whereas VBNC cells retained high amounts of rRNA and enzymatic activity [29]. In a milder environment (non-fermented media at neutral pH), far fewer cells entered the VBNC state, indicating that low pH stress was the cause of cell culturability in yogurt. Importantly, it was reported that, under favorable environmental conditions, such as pH neutralization or the provision of anerobic nutrient-rich media, VBNC Bifidobacteria can potentially resuscitate and resume growth [28]. Encapsulation and matrix optimization could partially mitigate VBNC entry in *Bifidobacterium* in dairy matrixes; however, resuscitation steps in enumeration protocols are still essential for accurate CFU enumeration [30,31]. A recent review focused on *Bifidobacterium* viability in dairy applications highlights that low pH and oxygen are key VBNC state drivers and recommends neutralization and anerobic pre-incubation to allow for cell resuscitation before plating [32].

### 2.4. Lacticaseibacillus rhamnosus GG (High-Pressure Processing Injury)

Exposure of *Lacticaseibacillus rhamnosus* GG to high hydrostatic pressure (100–600 MPa) results in sublethal cellular injury, triggering the VBNC state. While these cells cannot form colonies on agar plates (CFU method), their viability and metabolic activity were confirmed through the detection of residual esterase activity using flow cytometry [33,34,35]. The observation that colony recovery significantly increased on agar supplemented with the H_2_O_2_ scavenger, sodium pyruvate, indicates that oxidative damage contributes to the high-pressure induced VBNC state [12,36]. Pyruvate has a similar role to catalase in mopping up residual ROS/peroxides generated by the high-pressure stress, aiding cell culturability recovery [12]. Furthermore, these VBNC cells often display delayed colony appearance, necessitating extended incubation periods on agar plates to account for their slow resuscitation kinetics, as they do not undergo immediate exponential growth [36].

### 2.5. Evidence for VBNC in Encapsulated Probiotics

Recent studies indicate that encapsulation influences not only culturability but also the distribution of cells between culturable and VBNC states and additionally reduces the extent of cell death under stress. For example, spray-dried *L. rhamnosus* GG and *L. plantarum* 299v exhibited higher total viability when measured by metabolic activity (MTS assay) than by plate counts, suggesting that encapsulation matrices protected a subpopulation of cells that entered a VBNC state rather than undergoing cell death [22]. Similarly, microencapsulation of *Limosilactobacillus reuteri* DSPV002C has been shown to reduce the transition into a VBNC state and to limit cell death, thereby preserving overall functional viability [37]. Encapsulation mitigates stresses (e.g., acid, bile, oxidation, desiccation) that induce the VBNC state and cell death of probiotics through two mechanisms. The primary mechanism is a physical diffusion barrier created by the encapsulating material. For instance, gelatin in alginate–gelatin blends fills pores to improve acid buffering, while chitosan coatings provide a dense protective layer [38]. Secondly, the internal matrix, which can incorporate protective polymers or carbohydrates [39] has a protective effect.

**Table 1:** Case studies of VBNC induction and resuscitation of LAB in foods and beverages.

LAB Strain	Stress Condition	VBNC Evidence	Resuscitation Method	Key Observation	References
*Levilactobacillus brevis*	Cold storage and hop acids (beer)	No CFU on routine agar; viability and spoilage traits retained	Catalase supplemented MRS agar (1000 U/mL)	CFU recovered only on repair medium; spoilage function preserved	[23]
*Lactiplantibacillus plantarum*	Months at 4 °C or ≥30 beer passages	Metabolically active yet non-culturable on standard agar	Catalase supplemented agar	Recovery only with catalase from VBNC pool; oxidative stress implicated	[24]
*Fructilactobacillus lindneri* (formerly *Lactobacillus lindneri*)/*Lactobacillus acetotolerans*	Beer and cold stress	VBNC subpopulation; routine agar negative	Catalase supplemented agar	Culturability restored; spoilage retained; taxonomy updated	[25,26]
*Lacticaseibacillus paracasei* Zhang	Low temperature/acidic dairy matrices	VBNC transcriptome; flow-sorted viable cells	Nutrient-rich MRS	Extended lag before CFU rebound; minimal media ineffective	[19,20,27]
*Bifidobacterium* spp.	Acidic yogurt storage	Membrane-intact cells with high rRNA despite CFU loss	Neutral pH and anerobic recovery	VBNC cells revivable; plate counts undercount unless rescued	[30,31,32]
*Lacticaseibacillus rhamnosus* GG	High-pressure processing	Residual esterase activity by flow cytometry despite no CFU	Pyruvate/catalase agar; ≥72 h reads	CFU restored repair media; delayed colony emergence supports resuscitation	[33,34,35]

## 3. Reviving the VBNC State: Pathways and Conditions

### 3.1. Molecular Pathways Underlying VBNC Resuscitation

The resuscitation of VBNC bacteria is not a singular event but a complex process involving a coordinated reversal of the processes that induced the VBNC state (Table 2). Single-cell Raman spectroscopy further confirms broad molecular composition shifts between VBNC and recovered cells, reinforcing that resuscitation proceeds through a gradual and coordinated metabolic reactivation rather than immediate growth [19]. The key pathways involve sensing resuscitation signals, repairing critical cellular damage, and restarting core metabolic processes. Understanding these molecular triggers and repair mechanisms is crucial for developing strategies to actively recover the functionality of probiotic cultures.

#### 3.1.1. Sensing and Responding to Resuscitation Signals

The resuscitation of VBNC cells is initiated by the detection of favorable environmental conditions, which triggers a large-scale shift in gene expression to reverse the dormant state. A key mechanism for sensing resuscitation stimuli, such as nutrient availability or temperature shifts, is through two-component systems (TCSs). These systems detect specific environmental cues and trigger a transcriptional response that initiates recovery, a role established across diverse bacterial species [13]. This response is often synchronized at the population level through cell-to-cell communication. Quorum sensing (QS) molecules, such as autoinducer-2 (AI-2), accumulate until a critical threshold is reached, providing a density-dependent “wake up” call. This mechanism, which accelerates resuscitation in pathogens like *Vibrio* spp., may also be generalizable to LAB, despite observed strain-specific differences [13,40]. A related process involves Rpf (resuscitation promoting factor)-like enzymes. Although best characterized in *Actinobacteria*, some *Lactobacilli* possess genes for these hydrolytic enzymes. They function by cleaving the cell wall; the resulting muropeptides then act as signaling molecules that stimulate growth and resuscitation in neighboring cells [41]. Finally, a critical intracellular control point is the ribosome “sleep/wake” system, which allows for the rapid reactivation of protein synthesis upon sensing favorable conditions. During stress, ribosomes are inactivated to conserve energy. Key proteins like ribosome modulation factor (RMF) and ribosome-associated inhibitor A (RaiA) drive this hibernation by forming inactive 100 S dimers or pausing single ribosomes [42,43]. Alternatively, the hibernation factor Balon can block ribosomes mid-translation [44,45]. This protection from degradation ensures translational machinery is preserved and can be quickly restarted to fuel the resuscitation process.

#### 3.1.2. Repairing Cellular Damage

A critical prerequisite for resuscitation is the repair of damage accumulated during dormancy, with the reversal of oxidative stress being a particularly key step.

Oxidative stress repair: The scavenging of reactive oxygen species (ROS) is essential for restoring macromolecular synthesis and culturability. Compounds like sodium pyruvate play a dual role: they function as direct antioxidants and as metabolizable nutrients. This nutrient function is often mediated by sensory systems like the BtsSR–BtsT two-component system, which links environmental cues to nutrient transport and metabolism [12]. Food-relevant studies confirm that a combination of redox relief and nutrient upshift can effectively trigger the resuscitation of VBNC cells back to culturability without altering total viability [6,12].

DNA repair: Central to recovery is the rapid upregulation of genes involved in DNA repair pathways. The RecA protein, a master regulator of the SOS response and homologous recombination, is crucial for mending the double-stranded DNA breaks caused by oxidative and other stresses, enabling accurate genome replication to resume.

Evidence from multi-omics: Transcriptomic analysis of VBNC *Lactobacillus paracasei* Zhang strain reveals a survival-focused profile: the upregulation of molecular chaperones and stress response genes alongside the downregulation of translation and ATP-synthase genes, consistent with a state of energy conservation and macromolecular protection [20]. Subsequent metabolomics of resuscitating cells show a corresponding increase in protective metabolites and energy substrates, facilitating redox buffering and protein re-folding before cell division can restart [27]. Metabolomics in the same strain reveal increased pools of protective and refueling metabolites during recovery, aligned with redox buffering and protein-folding restoration before division resumes [27].

#### 3.1.3. Restoration of Metabolism

Finally, cells exiting a VBNC state must restore the pathways for growth and replication. Genes involved in glycolysis, ATP synthesis, and the biosynthesis of amino acids, nucleotides, and cofactors reactivate [4,6,46]. Peptidoglycan synthesis and cell division genes involved in cell wall biosynthesis must be expressed to allow for the first division, which is the ultimate proof of successful resuscitation [35,47].

**Table 2:** VBNC resuscitation: triggers and mechanisms.

Phase	Trigger	Mechanistic	Evidence
Sensing and Responding	Quorum signals (AI-2)	Community-level cues synchronize dormancy exit and accelerate recovery.	AI-2 promotes VBNC resuscitation in *Vibrio* models; food-micro reviews suggest a potential role in LAB resuscitation [13].
	Nutrients upshift	Replenishes ATP and biosynthetic precursors, reversing energy conservation programs and initiating growth.	The *BtsSR–BtsT* pyruvate-sensing/transport system links nutrient detection to resuscitation cues [46].
Repairing Cellular Damage	Redox relief (pyruvate, catalase)	Scavenges reactive oxygen species and restores DNA/protein synthesis before cell division resumes.	Pyruvate rescues VBNC cells and restores macromolecular synthesis; catalase similarly relieves oxidative blocks [11,41,46].
	Peptidoglycan remodeling (Rpf)	Low-level muralytic activity generates a cell-wall–derived “wake” signal and facilitates cell-cycle re-entry.	Rpf increases culturability in Gram positives and is reviewed as a resuscitation-promoting enzyme [42,43].
	Ribosome reactivation	Disengagement of hibernation factors frees ribosomes for translation after stress release.	Classical RaiA/RMF/HPF models plus the Balon–EF-Tu pathway [44,45] expand known “wake” mechanisms.
5 Restoration of Metabolism	Programmed stress repair (omics in LAB)	Coordinated upregulation of chaperones/stress pathways and downregulation of translation/ATP synthase during VBNC; metabolite rebounds during recovery.	*Lacticaseibacillus paracasei* Zhang shows transcriptomic and metabolomic transitions consistent with a repair-first growth-second model [20,27].

### 3.2. Chemical and Biological Factors Enhancing Resuscitation

Since the earliest nutrient-rich formulations such as peptone agar, bacterial cultivation media have evolved from simple recipes to specialized formulations with defined nutrients and growth factors. LAB, being nutritionally fastidious and lacking complete biosynthetic pathways, require complex media such as de Man, Rogosa, and Sharpe (MRS), which incorporate peptides, carbohydrates, minerals, and selective agents like acetate or citrate to favor LAB over competitors. Even so, strain-specific demands often necessitate modified or enriched formulations, which are often proprietary. These challenges are amplified for probiotic bacteria in a VBNC state, as their stringent nutritional requirements complicate both resuscitation and enumeration. To address this, researchers have explored supplementing growth media with amino acids, culture supernatants, or host cells, although such strategies remain inconsistent across species and even within repeated trials.

The primary and best-documented strategy for resuscitating VBNC probiotics involves alleviating oxidative stress, a major driver of VBNC induction, by antioxidants that scavenge harmful reactive oxygen species (ROS).

Catalase: This ROS-scavenging enzyme is highly effective at resuscitating VBNC cells by degrading peroxides and restoring macromolecular synthesis [12,23,24,25]. Its efficacy is well-established in food-relevant contexts; for example, supplementing agar with catalase restored the culturability of *L. brevis* and *L. plantarum* rendered VBNC by beer storage conditions [24,25]. Furthermore, its success in reviving *R. solanacearum* underscores that ROS relief is a universal resuscitation mechanism not limited to lactic acid bacteria [41].

Sodium pyruvate: This molecule functions both as a direct antioxidant and a metabolizable nutrient source. It promotes the recovery of peroxide-injured cells and is widely used as a repair supplement [11,12]. Its uptake via the BtsSR–BtsT two-component system links environmental sensing to nutrient transport and metabolism, allowing pyruvate to act as both a chemical scavenger of ROS and a nutrient cue that signals favorable conditions for awakening [46]. The combined supplementation of media with pyruvate and catalase is a well-documented practical mechanism to resuscitate cells exposed to sublethal oxidative stress [11,41].

Other fields of microbiology deploy further strategies for VBNC enumeration; for example, research in environmental microbiology has revealed that resuscitation promoting factors (Rpfs), secreted proteins originally identified in *Micrococcus luteus*, can effectively enhance the growth of soil bacterial species, including those hitherto uncultured [47]. Similarly, principles from medical microbiology show that spent media or purified Rpf proteins can resuscitate pathogens like *Vibrio cholerae* by supplying essential “wake-up factors” or growth stimulants [48]. The detergent Tween-80 was shown to help resuscitate *E. coli* VBNC cells, likely by altering membrane fluidity to facilitate nutrient uptake [49]. By adapting these insights from other fields, novel approaches can be developed to improve the enumeration of probiotic bacteria.

### 3.3. Nurturing Environments: Benefits of Prolonged Resuscitation in Liquid Media

An initial pre-enrichment step in a non-selective nutrient rich liquid medium is a highly effective step in the resuscitation of VBNC cells. A pre-enrichment broth is a liquid medium designed not for rapid robust growth but to gently repair sublethally injured cells and reverse the VBNC state before they are plated onto a solid selective medium. This “repair then select” approach provides cells with a suitable environment to repair macromolecular damage, to restart metabolic pathways, and to replenish energy levels without the stress of competition or the inhibitors present in selective agars [50]. This principle is well-established in classical microbiology, where formats like agar underlay/overlay are used to recover sublethally injured cells by offering a brief non-selective phase before exposure to selective agents [50]. This improved recovery is attributed to the greater accessibility of nutrients and redox-balancing compounds (e.g., pyruvate, catalase) in liquid media, which minimize oxidative stress and facilitate repair [9]. For oxygen-sensitive probiotics like *Bifidobacterium*, conducting both the pre-recovery and subsequent plating under anerobic neutral-pH conditions is essential, often converting an apparent absence of colonies into quantifiable CFU counts [30]. Valid evidence for true resuscitation, as opposed to the growth of a few survivors, is a significant increase in CFU counts following pre-recovery while the total viable population, measured by culture-independent methods (e.g., flow cytometry), remains constant [33].

## 4. Discriminating VBNC Resuscitation from Cell Proliferation Growth: Methodological Approaches

The key challenge in VBNC studies is to demonstrate that increases in culturability arise from the true resuscitation of VBNC cells rather than the growth of a minor culturable subpopulation [32,33,51]. Conclusive evidence therefore requires a multifaceted approach, which contrasts the distinct molecular and metabolic mechanisms underlying VBNC reversal from cell growth.

### 4.1. Culture-Independent Quantification of Total Viability

A critical first line of evidence requires demonstrating a constant number of total viable cells via a culture-independent method, such as flow cytometry per ISO 19344/IDF 232, alongside any rising CFU counts. This confirms that any increase in culturability is not due to population growth. Culture-independent assays are essential for detecting VBNC cells that remain metabolically active despite lacking culturability.

Flow cytometry (FC) provides an accurate quantification of live, damaged, and dead subpopulations within minutes. The ISO standard 19344/IDF 232 standardizes flow cytometric enumeration of lactic cultures and probiotics [52]. In mixed probiotic products, FC typically reports higher total viable counts than CFU under stress, indicating VBNC fractions that plate counts miss [17].

Live/dead staining and gating with fluorescent nucleic acid dyes such as SYTO 9 and propidium iodide enable the rapid classification of membrane-intact versus compromised cells for culture-independent viable enumeration [17]. Comparative studies in probiotic blends show that live/dead FACS quantification delivers greater accuracy, precision, and time-to-result than CFUs, particularly when cells are stressed or aggregated [17]. Operationally, a resuscitation signature is observed when total viable events by FACS remain approximately constant while CFUs rise only after repair steps.

For species/strain resolution, Flow-FISH integrates rRNA probes in flow cytometry to enumerate viable cells in 2–2.5 h, overcoming the strain ambiguity of bulk cytometry in blends of different species [14,52]. A recent study demonstrates that Flow-FISH and live/dead FC provide higher viable cell counts than plate counting (i.e., plate counting underestimates viable cells), for Gram-positive probiotic products and mixtures [52]. Combinations of PMA-qPCR/dPCR and Flow-FISH can support the cross-validation of strain assignments and strengthens VBNC interpretation in multi-strain formulations [52].

Impedance flow cytometry (IFC) methods can be used to classify viability states from single-cell electrical signatures without stains, and have been reported to differentiate live versus inactivated bacteria in proof-of-concept studies [53]. Follow-up work indicates that IFC performance depends on medium conductivity and population stress history, highlighting optimization needs before routine use [54].

Operational criteria and rationale. To distinguish true VBNC resuscitation from ordinary growth, quantitative thresholds should be applied to reduce false positives caused by counting variability and to ensure the method is practical for routine quality control (QC). For total viable counts, levels should be within ±0.2 log_10_ before and after the resuscitation period [52]. A CFU increase of ≥0.5–1.0 log_10_ should be observed under resuscitation conditions, while the change should remain <0.2 log_10_ in a rich control medium, lacking resuscitation aids. These criteria ensure that the observed increase exceeds typical plate count uncertainty. This approach is consistent with previous reports showing that the relief of oxidative injury and mild pre-recovery treatments are essential for restoring culturability in lactic acid bacteria and *Bifidobacterium* spp. [11,12,23,24,25,26,27,28,36].

### 4.2. Establishing Condition-Specific Resuscitation

Secondly, it should be shown that CFU increases only occur under specific “repair” conditions designed to reverse the original injury and not in a standard nutrient-rich control medium. This condition-dependent revival is central to true VBNC reversal. It should be demonstrated that the increase in culturability (CFUs) occurs only when the specific “resuscitation signal” or “repair condition” is present (e.g., + resuscitation supplement(s)) and does not occur in a standard nutrient-rich control medium that supports the growth of any already-culturable cells. The revival is dependent on a specific environmental cue.

### 4.3. Supporting Phenotypic Signatures of VBNC Exit

Thirdly, capturing phenotypic signatures unique to cells recovering from the VBNC state, such as delayed colony appearance (e.g., ScanLag) and shifts in colony morphology [51]. This includes the direct viable count (DVC) assay, which uses division inhibitors to allow metabolically awakening cells to elongate without dividing, physically separating resuscitation from growth [55]. The method involves the inhibition of bacterial cell division while simultaneously supplying a mild nutrient stimulus, enabling metabolically active cells to enlarge or elongate without undergoing multiplication, thereby allowing resuscitation events to be distinguished from cell growth. This approach is particularly relevant for VBNC populations, where the addition of a suitable growth inhibitor, such as chloramphenicol at an optimized concentration, to the medium can prevent cell division while allowing metabolic repair. Under such conditions, the subsequent recovery of CFU after inhibitor removal or during specific resuscitation treatments provides strong evidence that culturability is restored via VBNC resuscitation rather than by growth and proliferation of surviving cells [56].

Furthermore, culture-based kinetic analyses can reveal characteristic VBNC resuscitation signatures. A VBNC exit typically presents as late colony emergence and extended lag phases, measurable by automated colony appearance analysis such as Scan Lag [51]. A constant cytometric quantification at different time points with delayed CFU appearance indicates resuscitation, whereas early CFU gains with rising totals suggest survivor proliferation [10,36]. Acidification assays, such as pH monitoring in standard media can outline sublethal injuries in probiotic populations [2]. Isothermal microcalorimetry (IMC) measures heat flow from metabolizing cells and sensitively distinguishes viable activity in complex matrices, including probiotics [53,54]. IMC case studies in food microbiology reveal lag phase extensions and stress effects faster than CFU-based methods [2].

### 4.4. Integrated Application in Probiotic Quality Control

Modern probiotic quality control increasingly integrates both culture-based and culture-independent metrics, reporting CFU alongside total viable counts so that VBNC fractions are not underestimated [2]. As standardized in ISO 19344, flow cytometry measurements are routinely applied in food laboratories [52]. Despite these advances, regulatory labels in many jurisdictions still require end of shelf life CFU stability, and pharmacopeial guidance recommends validating alternative methods in parallel, enabling dual-metric specifications in practice [2,5].

### 4.5. Analytical Perspective: Strengths, Limits, Evidence, and Implications

The framework described above is well-suited for quality control implementation because its quantitative criteria are in alignment with routine counting variability and are consistent with the ISO-standardized cytometric enumeration of total viable counts (TVCs) [52]. From a practical standpoint, the approach offers several advantages: it is compatible with conventional media and supplements such as catalase and pyruvate; the pre-recovery step is short and low in workload; and clearly defined observation windows (48–72 h) allow the detection of delayed colonies that would otherwise be classified as negative. These attributes make the protocol feasible for industrial or regulatory QC environments where throughput and reproducibility are key.

Nevertheless, the framework also presents limitations that affect its general applicability. Strain- and matrix-dependent growth behavior can influence recovery dynamics, and the requirement for cytometry expertise to quantify total viable counts may constrain its adoption in more basic laboratories. Furthermore, slow-resuscitating populations can extend turnaround times, potentially limiting use in rapid release QC workflows. Evidence from multiple food-related studies demonstrates that catalase or pyruvate supplementation relieves oxidative injury and promotes resuscitation in *Lactobacillus* [23,24,25,26] and *Bifidobacterium* [30,31,32] species, with late colony emergence patterns consistent with VBNC exit [11,12,36] and ScanLag-type appearance time shifts [51]. However, these responses are often species- or strain-specific, and food matrices such as dairy or beverage systems differ substantially in redox potential, pH, and oxygen transfer—factors that affect the reproducibility and transferability of results.

Current validation gaps include limited inter-laboratory consistency and insufficient evaluation in complex product matrices. These could be addressed through collaborative ring trials and standardized reporting of appearance time metrics. The alignment of targets between AFU/TVC and resuscitated CFU counts would strengthen comparability across methods. Flow cytometry-based measurements of AFU/TVC should serve as a complementary metric to CFU, which remains the regulatory benchmark. Documenting repair conditions, thresholds, and appearance time data enhances transparency and reproducibility, thereby reducing the risk of underestimating VBNC fractions while keeping compliance anchored to CFU-based enumeration. Collectively, this analytical framework provides a practical bridge between emerging VBNC detection science and the operational needs of probiotic quality control.

## 5. A Dual Measurement Strategy for Accurate Probiotic Potency Assessment

In this section, a two-step dual-metric workflow is proposed that integrates standardized flow cytometry with resuscitation-enhanced CFU enumeration. This approach ensures that samples are assessed both for total viable cells and for recoverable culturable cells under defined resuscitation conditions. Practical media formulations and incubation parameters supplements, concentration ranges, pH, atmosphere, and timing are specified below and in Section 3 to support reproducible application across laboratories. The first step is to determine the total viable population, as measured by AFU. The overall viable population is quantified by flow cytometry according to ISO 19344 [52], distinguishing live, damaged, and dead fractions. This provides a rapid standardized estimate of total viable cells that serves as a benchmark for subsequent CFU comparison.

Secondly, it is proposed to determine resuscitation-enhanced CFU enumeration. Samples undergo a pre-recovery incubation in liquid nutrient broth adjusted to pH 6.8–7.2 for 2–24 h at 30–37 °C. Anerobic or microaerophilic conditions are applied for oxygen-sensitive taxa (e.g., *Bifidobacterium* spp.). After this recovery phase, cultures are plated on agar supplemented with catalase (>100 U/mL) and/or sodium pyruvate (>0.1% *w*/*v*). Plates are incubated for 48–72 h to capture delayed colony formation typical of VBNC resuscitation. These parameters are compatible with routine QC media and have been shown to promote the repair of oxidative injury without introducing selective bias.

Acceptance criterion. Following validated resuscitation, an absolute difference of |AFU − CFU| ≤ 0.5 log_10_ indicates a satisfactory agreement between total viable and culturable counts. Larger discrepancies signify a substantial VBNC or injured subpopulation, prompting the investigation of manufacturing, storage, or formulation factors that may impair cell recovery.

This standardized parameterized workflow minimizes ambiguity and provides an operationally practical method to detect and quantify VBNC fractions in probiotic quality assessment.

Accurately quantifying probiotic levels is paramount for ensuring efficacy, regulatory compliance, and consumer trust. To address the systematic underestimation of viability caused by the VBNC state, a standardized dual measurement strategy is recommended. This workflow integrates culture-independent quantification with advanced culture-based methods to capture the total viable population, including VBNC cells, thereby providing a more comprehensive and accurate measure of a product’s true potency.

The recommended approach is to measure (1) the total viable count via standardized flow cytometry and (2) resuscitation-enhanced culturability.

Flow cytometry, conducted according to the international standard ISO 19344|IDF 232, quantifies the total number of membrane-intact metabolically active cells. The measurement, reported as active fluorescent units (AFUs) or total viable count (TVC), captures the entire viable population, including culturable, injured, and VBNC cells, and serves as the benchmark for true viability [51,56,57,58,59,60].

Resuscitation-enhanced culturability. To reconcile total viability with the regulatory requirement of colony-forming units (CFUs), plate samples onto supplemented and resuscitation-promoting media (as detailed in Section 3). The supplemented growth media is specifically designed to reverse sublethal injury and resuscitate VBNC cells, thereby yielding a CFU count that more accurately reflects the true number of viable cells capable of replication [9,50].

This dual measurement approach establishes a principle of bioequivalence for probiotic quantification. Analogous to pharmaceuticals, where a generic drug must demonstrate equivalence to the original, the CFU count obtained after resuscitation (Step 2) should be validated against the total viable count (Step 1). For a high-quality product with minimal VBNC cells, the AFU and CFU values will be equivalent. A significant divergence, where AFU > CFU, indicates a substantial VBNC population and signals that the product’s viability has been compromised by processing or storage stresses, even if the initial CFU count was high.

Implementing this strategy has several benefits, including enabling manufacturers to prevent overdosing by formulating products based on accurate viability data, avoiding the excessive overdosing currently needed to meet label claims at the end of shelf life. It also strengthens regulatory compliance and consumer trust by providing a more scientifically robust and transparent assessment of probiotic potency that aligns with the latest microbiological developments.

This workflow relies on routine media and inexpensive supplements. However, practical constraints can arise from strain/matrix variability, with the potential need for adjustment and optimization of critical method parameters. These include media pH, time frame for pre-recovery incubation, nutrient and supplement concentrations in media, and the need for stricter anaerobiosis in some taxa.

## 6. Conclusions

This review consolidates and critically evaluates the current evidence on VBNC detection and resuscitation in lactic acid bacteria and probiotic products, providing a quantitative testing framework to distinguish true resuscitation from growth. By defining objective thresholds and practical recovery conditions, it advances beyond descriptive summaries to deliver a usable basis for method standardization. Coupling standardized flow cytometric AFU assessment with resuscitation-enhanced CFU enumeration enables more accurate potency estimation, tighter alignment of labeling with actual viable cell content, and reduced reliance on overdosing to compensate for undetected VBNC fractions.

The proposed dual-metric workflow offers clear practical advantages for quality control and regulatory compliance but also faces constraints related to strain- and matrix-specific effects, anerobic handling requirements, and access to cytometry expertise. Future priorities include inter-laboratory ring trials, establishment of consensus quantitative cut-offs for AFU–CFU agreement, and validation across representative food and supplement matrices. Collectively, these steps would support harmonized standards for probiotic viability testing, bridging scientific rigor with regulatory applicability.

## Data Availability

The original contributions presented in this study are included in this article. Further inquiries can be directed to the corresponding authors.
